# Large-scale analysis reveals racial disparities in the prevalence of ADHD and conduct disorders

**DOI:** 10.1038/s41598-024-75954-5

**Published:** 2024-10-24

**Authors:** Noha Shalaby, Sourav Sengupta, Jamal B. Williams

**Affiliations:** 1 Department of Psychiatry State University at Buffalo, Jacobs School of Medicine and Biomedical Sciences, 875 Ellicott St., Buffalo, NY 14203 USA; 2 Erie County New York State Public Health Corps., Buffalo, NY USA

**Keywords:** ADHD, Epidemiology

## Abstract

The primary purpose of this study is to highlight trends in the prevalence of attention deficit/hyperactivity disorders (ADHD) and conduct disorders (CD) between non-Hispanic White and non-Hispanic Black populations and identify potential diagnostic disparities between these groups. De-identified electronic health record data on the TriNetX platform of patients diagnosed with ADHD, CD, or both between January 2013 and May 2023 from 50 healthcare organizations in the US were used to investigate racial and sex disparities in the prevalence of ADHD and CD diagnoses. With a cohort of 849,281 ADHD patients and 157,597 CD patients, non-Hispanic White individuals were ~ 26% more likely to receive ADHD diagnosis and ~ 61% less likely to be diagnosed with CD than non-Hispanic Black individuals. The mean age of diagnosis of ADHD was over 8 years older for White patients than for Black patients, with a disproportionately higher number of White patients diagnosed in adulthood, compared to a comparatively negligible number of Black patients diagnosed with ADHD in the same age group. Additionally, Black females were the cohort least likely to be diagnosed with ADHD, while White females were the cohort least likely to be diagnosed with CD. Race disparities exist between Black and White populations, and sex disparities exist within each population. More information is needed to determine contributors to these differences, although implicit biases and systemic racism may be key contributing factors. Presenting evidence and increasing awareness of culturally relevant diagnoses can reduce unconscious bias and move toward more informed and objective psychiatric evaluations.

## Introduction

Attention-deficit/hyperactivity disorder (ADHD) and conduct disorder (CD) are behavioral conditions that affect approximately 10% and 3% of children in the United States, respectively^1,2^. However, the subjective nature of symptom assessment and the apparent overlap in behavioral manifestations between these disorders pose a significant risk for misdiagnosis^3,4^. This issue is further complicated by the cultural context in which symptoms are evaluated, leading to potentially inappropriate categorization of behaviors, and are likely to have negative downstream effects in discrepancies in diagnostic prevalence of ADHD and CD across racial and ethnic groups^5–7^.

Both ADHD and CD have high societal and economic costs. ADHD persists into adulthood in about one-half to two-thirds of cases^8–11^. Moreover, despite being characterized as a neurodevelopmental disorder in childhood, there is a recent recognition of adult ADHD cases^11,12^. Current studies investigate whether ADHD in adults stems from missed childhood symptoms or comorbid mental health disorders rather than a distinct ‘late onset’ presentation^10–13^. As for CD, about 50% of individuals have a remission of symptoms in adulthood, while the rest frequently grow up to have a high risk of substance abuse, display criminal behaviors, or develop personality disorders^2,14^.

There is growing evidence that minority populations are less likely to be diagnosed with ADHD and less likely to take medication for ADHD compared to non-Hispanic White populations^5,6,15^. Additionally, Black and Hispanic children are more likely to be diagnosed with CD than non-Hispanic White children^5,6^. Despite this growing evidence, these findings are often based on limited sample sizes and do not directly compare ADHD and CD diagnostic prevalence. Previous studies have also not investigated diagnostic prevalence at the subtype level. Our study leverages a large-scale dataset of de-identified electronic health records, which includes ADHD and CD subtypes, to provide a more comprehensive analysis of these disparities. By comparing the prevalence of both ADHD and CD diagnoses, we aim to offer new insights into how diagnostic biases and cultural contexts may influence the categorization of these behavioral conditions. We hypothesize that non-Hispanic Black populations exhibit a lower likelihood of ADHD diagnosis, and this difference in diagnosis rates between Black and White populations may be linked to an overrepresentation of Oppositional Defiant disorder (ODD) and CD diagnosis in Black communities. Furthermore, we suspect that there is also consensus that males are two to four times more likely to be diagnosed with neurodevelopmental disorders than females^5,16^. Although this sex discrepancy may be related to the interplay between biological and societal factors, there is concern over diagnostic bias contributing to the underdiagnosis of females with neurodevelopmental disorders^16^.

## Methods

### Data collection

This retrospective study was conducted using the TriNetX Research Network, which provides access to approximately 117 million anonymized patient electronic medical records from nearly 80 healthcare organizations across 4 countries. We first acquired data from patients diagnosed with ADHD (ICD-10 code F90), totaling 1,659,318 records. The second cohort acquired were patients with a CD diagnosis (ICD-10 code F91), with 422,625 patient records. These data were collected on June 5th, 2023. Our primary focus was on patient records within the US, consisting of approximately 96 million patients from 57 healthcare organizations.

### Study design

With the high prevalence and increased knowledge of ADHD worldwide, the American Psychiatric Association’s (APA) 2013 update to the Diagnostic and Statistical Manual of Mental Disorders (5th ed.; DSM–5)^17^ included revised criteria for the ADHD diagnosis^18^. Consequently, the annual prevalence of ADHD and CD diagnoses in our cohort data from 2000 to 2022 showed a steep increase in cases in recent years, particularly following 2013. Therefore, only patients with an initial diagnosis after 2013 were counted in each cohort. Using patient demographics, all patient records from outside the US or patients whose location was marked as “Unknown” were excluded. The focus was narrowed down to two groups of interest, a non-Hispanic White group, and a non-Hispanic Black/African American group. Each of these groups was further divided by sex into males and females.

ICD-10 codes were used to stratify each cohort by presentation, with some individuals falling under multiple presentations. The ADHD cohorts were divided into ADHD, predominantly inattentive type (F90.0), ADHD, predominantly hyperactive type (F90.1), ADHD, combined type (F90.2), Other ADHD (F90.8), and Unspecified ADHD (F90.9). In this study, other and Unspecified ADHD were combined under the label Unspecified ADHD. The CD cohorts were divided into Oppositional Defiant Disorder (ODD, F91.3), Childhood-onset CD (F91.1), Adolescent-onset CD (F91.2), Other CD (F91.8), and Unspecified CD (F91.9). In this study, other and Unspecified CD were combined under the label Unspecified CD.

Each patient’s age of diagnosis (AoD) was determined by subtracting the year of the first recorded diagnosis of the disorder from the year of birth.

### Analysis and statistics

The reference population numbers for non-Hispanic White, non-Hispanic Black, and the total male and female populations were derived using queries on the TriNetX platform.

To compare each diagnosis and its associated presentations between the different race and sex cohorts, prevalence ratios were calculated. For example, the number of non-Hispanic Black individuals presenting with ADHD was divided by the total number of non-Hispanic Black individuals in the dataset to determine the prevalence of ADHD in the non-Hispanic Black population. To compare with the prevalence of the diagnosis in the non-Hispanic White population, the ADHD prevalence in the non-Hispanic Black population was divided by the ADHD prevalence in the non-Hispanic White population to get the prevalence ratio. The same was done with the non-Hispanic White ADHD population, resulting in a value of 1 for the White ADHD population and a value relative to 1 for the Black ADHD population. This process was repeated for each presentation of ADHD and CD in each race and sex cohort, comparing their prevalence in the Black population to the White population, and in the Black Male, Black Female, and White Female populations to the White Male population (Figs. S1 and S2).

Fisher’s exact tests were used to determine the significance of the differences in the prevalence of each disorder and each disorder’s presentations across race and sex. The *p*-values resulting from the Fisher exact tests were adjusted for false discovery rate (FDR).

Student t-tests were used to determine the significance of the difference in the mean of the AoD between White and Black patients diagnosed with either ADHD or CD.

Chi-squared tests were used to determine the degree of association between race and sex in the prevalence of ADHD or CD diagnoses in a population by creating two-way contingency tables combining the race and sex distribution of each disorder.

All data processing, statistical analyses, and figure generation were conducted in R^19–22^.

### Ethics declarations

TriNetX, LLC, complies with the Health Insurance Portability and Accountability Act (HIPAA). The data reviewed is a secondary analysis of existing data, does not involve intervention or interaction with human subjects, and is de-identified per the de-identification standard defined in Section 164.514(a) of the HIPAA Privacy Rule. The process by which the data is de-identified is attested to through a formal determination by a qualified expert as defined in Section 164.514(b)(1) of the HIPAA Privacy Rule. This formal determination by a qualified expert was refreshed in December 2020.

This study only uses data collected from the *TriNetX Research* Network which contains data provided by healthcare organizations that allow the use of their data for scientific research and publications and warrant that they have all necessary rights, consents, approvals, and authority to provide the data to TriNetX under a Business Associate Agreement, so long as their name remains anonymous as a data source and their data are utilized for research purposes. This retrospective study is therefore exempt from informed consent by the University at Buffalo’s Institutional Review Board. The methods used in this study reveal no identifying information of either the subjects or the healthcare organizations.

## Results

Amongst US patients on the TriNetX platform, 32,489,776 were non-Hispanic White, including 17,111,169 (52.7%) females. The non-Hispanic Black population accounted for 8,702,848 individuals, 4,654,839 (53.5%) females. In the White cohort, 708,004 individuals (2.18%) were diagnosed with ADHD. Out of those, 313,138 (44.2%) were females. Additionally, 110,160 (0.34%) were diagnosed with ODD or CD, and 36,300 (33%) were females. In the Black cohort, 141,277 (1.62%) were diagnosed with ADHD, including 51,323 (36.3%) females and 47,437 (0.55%) were diagnosed with ODD or CD, including 17,047 (35.9%) females (Table S1). The years of birth of the patient population ranged from 1927 to 2022 with approximately 2% of the population recorded dead at the time of data collection. The regional distribution of the non-Hispanic Black cohort in the US was 27.8% from the Midwest, 13.7% from the Northeast, 55% from the South and 3.5% from the West, while the distribution of the non-Hispanic White cohort was 32.3% from the Midwest, 20.2% from the Northeast, 35.1% from the South and 12.4% from the West.

**Fig. 1:**
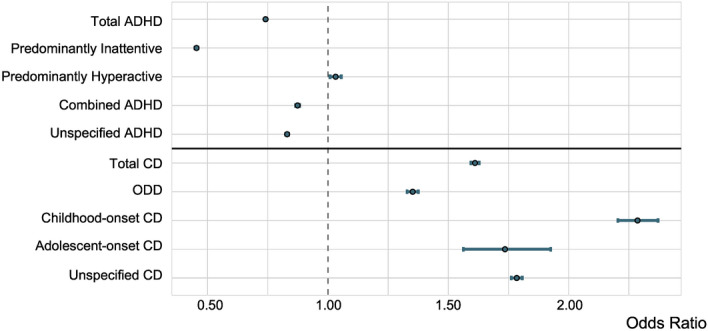
Prevalence of ADHD and CD between Black and White Individuals. Fisher’s exact test Odds Ratio and 95% confidence interval comparing the prevalence of ADHD and CD and their presentations in the Black population to the White population. ADHD; Black N = 141,277, White N = 708,004; CD; Black N = 47,437, White N = 110,160.

Figure 1 shows the odds ratio and 95% confidence interval resulting from Fisher’s exact test comparing the prevalence of each disorder and its presentations in the Black and White populations with the exact values and the statistical significance listed in Table 1. Apart from ADHD hyperactive type (ADHD-HT), ADHD diagnoses are significantly less prevalent in the Black population than in the White population. In the total ADHD cohort, a diagnosis in the Black population is 26% (OR 0.74; 95% CI 0.73–0.74; *P* < 0.0001) less prevalent than in the White population. ADHD inattentive-type (ADHD-IT) is 55% (OR 0.45; 95% CI 0.45–0.46; *P* < 0.0001) less prevalent in the Black population, while ADHD-HT is 0.03% (OR 1.03; 95% CI 1.01–1.05; *P* = 0.008) more prevalent in the Black population. For ADHD combined-type (ADHD-CT) and unspecified ADHD, a diagnosis is, respectively, 13% (OR 0.87; 95% CI 0.86–0.88; *P* < 0.0001) and 17% (OR 0.83; 95% CI 0.82–0.84; *P* < 0.0001) less prevalent in the Black population than in the White population.

**Table 1:** Fisher’s Exact Test comparing the prevalence of ADHD and its presentations and CD and its presentations between the Black and White populations.

	Number of black patients	Number of white patients	Odds ratio	95% confidence interval	FDR adjusted *p*-value
Total ADHD	141,277	708,004	0.74	0.73–0.74	< 0.0001
Predominantly Inattentive	24,047	197,246	0.45	0.45–0.46	< 0.0001
Predominantly Hyperactive	9,416	34,073	1.03	1.01–1.05	0.008
Combined ADHD	53,895	230,083	0.87	0.86–0.88	< 0.0001
Unspecified ADHD	107,660	482,871	0.83	0.82–0.84	< 0.0001
Total CD	47,437	110,160	1.61	1.59–1.63	< 0.0001
ODD	17,826	49,248	1.35	1.33–1.37	< 0.0001
Childhood-onset CD	4698	7673	2.28	2.21–2.37	< 0.0001
Adolescent-onset CD	530	1140	1.73	1.56–1.92	< 0.0001
Unspecified CD	34,590	72,490	1.78	1.76–1.81	< 0.0001

CD diagnoses are more prevalent in the Black population than in the White population. In the total CD cohort, a diagnosis in the Black population is 61% (OR 1.61; 95% CI 1.59–1.63; *P* < 0.0001) more prevalent than in the White population, while ODD is 35% (OR 1.35; 95% CI 1.33–1.37; *P* < 0.0001) more prevalent. Childhood-onset CD and adolescent-onset CD are, respectively, 128% (OR 2.28; 95% CI 2.21–2.37; *P* < 0.0001) and 73% (OR 1.73; 95% CI 1.56–1.92; *P* < 0.0001) more prevalent in the Black population while unspecified CD was 78% (OR 1.78; 95% CI 1.76–1.81; *P* < 0.0001) more prevalent than in the White population.

**Fig. 2:**
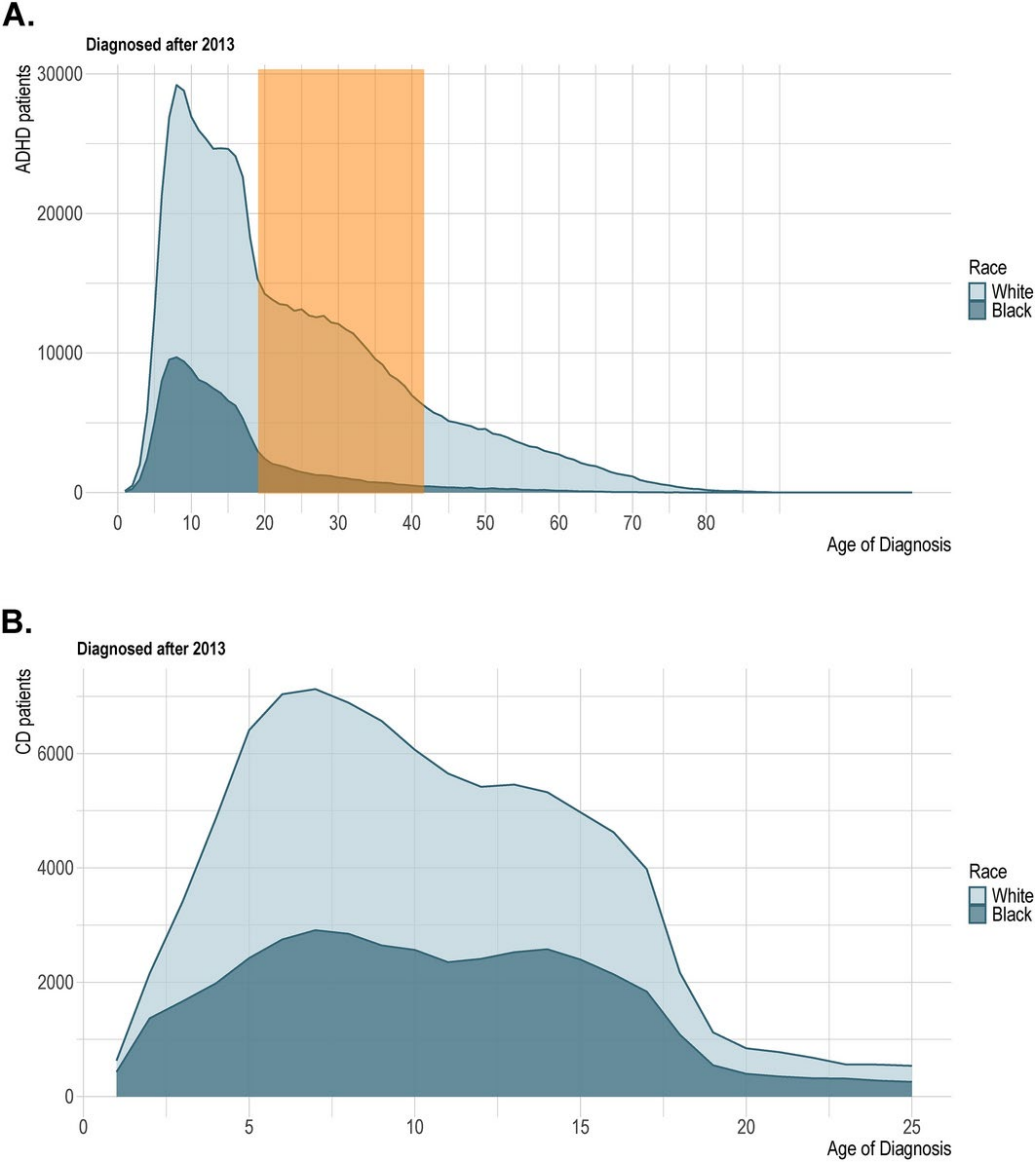
Age of Diagnosis Distributions. (**A**) Density plot of the AoD distribution of ADHD in Black and White patients, (**B**) Density plot of the AoD distribution of CD in Black and White patients.

The AoD for each cohort was calculated, and we discovered that the average ADHD AoD of White patients is 23.9, which is significantly higher than the average ADHD AoD of Black patients at 15.7 (95% CI 8.1–8.24; *P* < 0.0001), attributed to the relative increase in the diagnosis of White individuals between the ages of 18 and 40 years old (Table S2). Figure 2a highlights that the AoD distribution between Black and White individuals with ADHD are essentially the same in childhood, yet there is a substantial increase in the diagnostic prevalence of ADHD in White adults compared to Black adults. The AoD distribution of Black and White CD patients shows more parallel trends between the two groups (Fig. 2b).

**Fig. 3:**
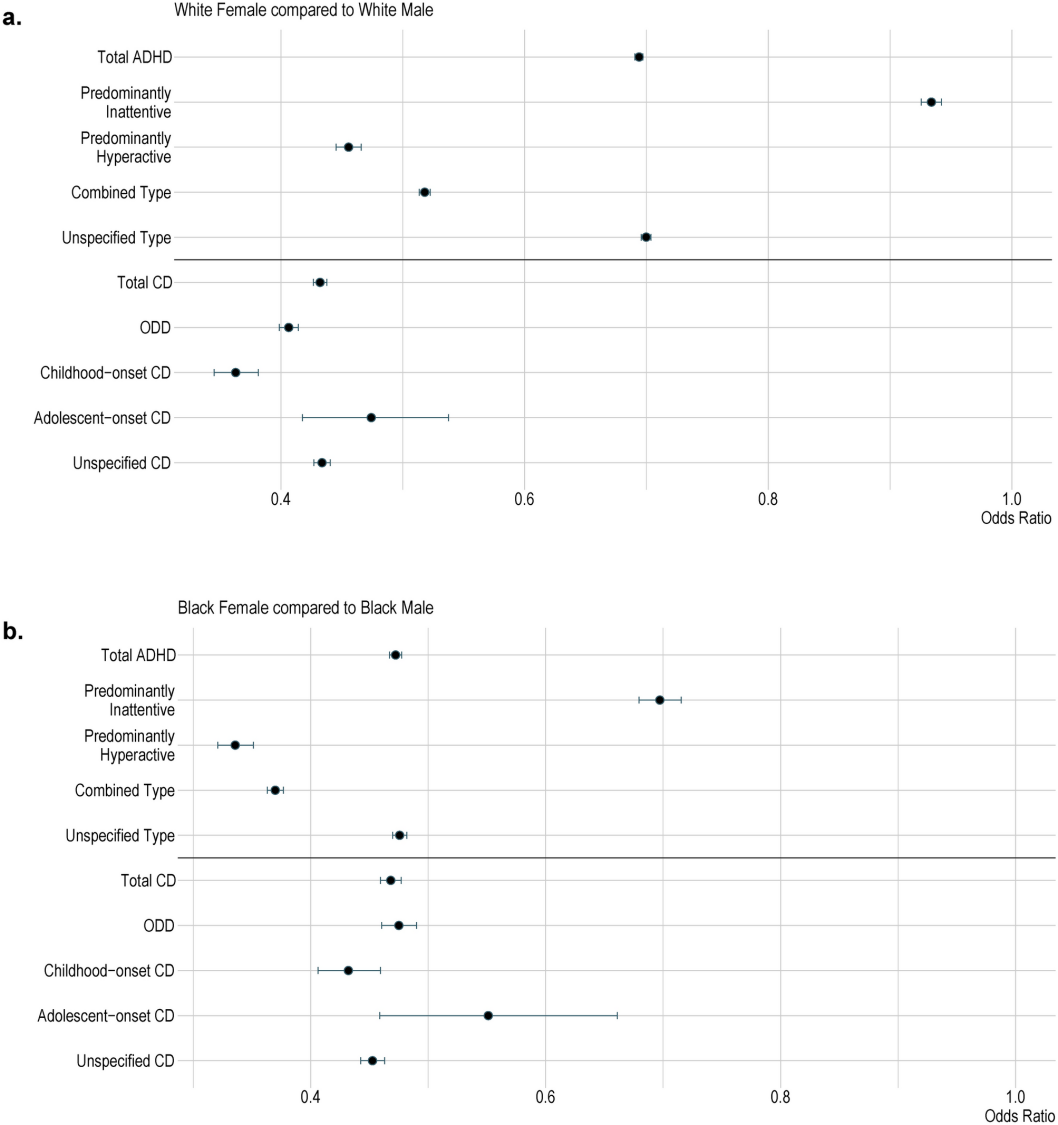
Sex Differences in ADHD and CD Prevalence within Black and White Populations. Fisher’s exact test Odds Ratio and 95% confidence interval of the prevalence of ADHD and CD and their presentations in (**a**) the White female population compared to the White male population and (**b**) the Black female population compared to the Black male population. ADHD; Black Female N = 51,323, Black Male N = 89,935, White Female N = 313,138, White Male N = 394,607; CD; Black Female N = 17,047, Black Male N = 30,381, White Female N = 36,300, White Male N = 73,837.

When considering sex differences, females have a lower diagnostic prevalence of ADHD and CD across Black and White populations (Tables 2 and 3). In Fig. 3a, the odds ratios indicate that White females are consistently less likely to be diagnosed with ADHD and its subtypes compared to White males, with similar patterns observed for CD and its presentations. Figure 3b shows that Black females also exhibit significantly lower odds of being diagnosed with both ADHD and CD compared to Black males, reinforcing the underrepresentation of females in these diagnoses across both racial groups.

**Table 2:** Fisher’s Exact Test comparing the prevalence of ADHD and its presentations in each of the Black Female, Black Male and White Female populations to their prevalence in the White Male population and within their race and sex group.

	Cohort	Number of patients	Odds ratio	95% confidence interval	FDR adjusted *p*-value
Total ADHD compared to White Male (N = 394,607)	Black female	51,323	0.41	0.41–0.42	< 0.0001
	Black male	89,935	0.88	0.87–0.89	< 0.0001
	White female	313,138	0.69	0.69–0.70	< 0.0001
Total ADHD compared to Black Male (N = 89,935)	Black female	51,323	0.47	0.46–0.48	< 0.0001
Total ADHD compared to White Female (N = 313,138)	Black female	51,323	0.60	0.59–0.61	< 0.0001
Predominantly Inattentive ADHD compared to White Male (N = 94,857)	Black female	10,790	0.37	0.36–0.37	< 0.0001
	Black male	12,953	0.53	0.52–0.54	< 0.0001
	White female	100,539	0.93	0.92–0.94	< 0.0001
Predominantly Inattentive ADHD compared to Black Male (N = 12,953)	Black female	10,790	0.69	0.68–0.72	< 0.0001
Predominantly Inattentive ADHD compared to White Female (N = 100,539)	Black female	10,790	0.39	0.38–0.40	< 0.0001
Predominantly Hyperactive ADHD compared to White Male (N = 22,345)	Black female	2680	0.39	0.37–0.40	< 0.0001
	Black male	6680	1.16	1.12–1.19	< 0.0001
	White female	11,558	0.45	0.44–0.46	< 0.0001
Predominantly Hyperactive ADHD compared to Black Male (N = 6680)	Black female	2680	0.34	0.32–0.35	< 0.0001
Predominantly Hyperactive ADHD compared to White Female (N = 11,558)	Black female	2680	0.85	0.82–0.89	< 0.0001
Combined ADHD compared to White Male (N = 143,452)	Black female	16,424	0.37	0.36–0.37	< 0.0001
	Black male	36,983	0.99	0.98–1.01	0.62
	White female	84,691	0.52	0.51–0.52	< 0.0001
Combined ADHD compared to Black Male (N = 36,983)	Black female	16,424	0.37	0.36–0.38	< 0.0001
Combined ADHD compared to White Female (N = 84,691)	Black female	16,424	0.71	0.70–0.72	< 0.0001
Unspecified ADHD compared to White Male (N = 264,589)	Black female	38,154	0.46	0.45–0.47	< 0.0001
	Black male	66,593	0.97	0.96–0.98	< 0.0001
	White female	211,128	0.69	0.69–0.70	< 0.0001
Unspecified ADHD compared to Black Male (N = 66,593)	Black female	38,154	0.48	0.47–0.48	< 0.0001
Unspecified ADHD compared to White Female (N = 211,128)	Black female	38,154	0.66	0.65–0.67	< 0.0001

**Table 3:** Fisher’s Exact Test comparing the prevalence of CD and its presentations in each of the Black Female, Black Male and White Female populations to their prevalence in the White Male population and within their race and sex group.

	Cohort	Number of patients	Odds ratio	95% confidence interval	FDR adjusted *p*-value
Total CD compared to White Male (N = 73,837)	Black Female	17,047	0.75	0.73–0.76	< 0.0001
	Black Male	30,381	1.59	1.57–1.62	< 0.0001
	White Female	36,300	0.43	0.42–0.44	< 0.0001
Total CD compared to Black Male (N = 30,381)	Black Female	17,047	0.47	0.46–0.48	< 0.0001
Total CD compared to White Female (N = 30,300)	Black Female	17,047	1.73	1.70–1.76	< 0.0001
ODD compared to White Male (N = 32,997)	Black Female	6,256	0.61	0.59–0.63	< 0.0001
	Black Male	11,017	1.29	1.26–1.32	< 0.0001
	White Female	15,233	0.41	0.40–0.41	< 0.0001
ODD compared to Black Male (N = 11,017)	Black Female	6256	0.48	0.46–0.49	< 0.0001
ODD compared to White Female (N = 15,233)	Black Female	6256	1.51	1.47–1.56	< 0.0001
Childhood-onset CD compared to White Male (N = 5,350)	Black Female	1567	0.94	0.89–1.00	0.07
	Black Male	3037	2.19	2.10–2.29	< 0.0001
	White Female	2203	0.36	0.34–0.38	< 0.0001
Childhood-onset CD compared to Black Male (N = 3037)	Black Female	1567	0.43	0.41–0.46	< 0.0001
Childhood-onset CD compared to White Female (N = 2203)	Black Female	1567	2.62	2.45–2.79	< 0.0001
Adolescent-onset CD compared to White Male (N = 712)	Black Female	200	0.91	0.77–1.06	0.26
	Black Male	304	1.65	1.44–1.89	< 0.0001
	White Female	383	0.47	0.42–0.54	< 0.0001
Adolescent-onset CD compared to Black Male (N = 304)	Black Female	200	0.55	0.49–0.66	< 0.0001
Adolescent-onset CD compared to White Female (N = 383)	Black Female	200	1.92	1.61–2.28	< 0.0001
Unspecified CD compared to White Male (N = 47,730)	Black Female	11,915	0.81	0.79–0.82	< 0.0001
	Black Male	21,987	1.78	1.75–1.81	< 0.0001
	White Female	23,528	0.43	0.42–0.44	< 0.0001
Unspecified CD compared to Black Male (N = 21,987)	Black Female	11,915	0.45	0.44–0.46	< 0.0001
Unspecified CD compared to White Female (N = 23,528)	Black Female	11,915	1.86	1.82–1.91	< 0.0001

Figure S3 further compares these odds ratios by focusing on Black females relative to White males (S3a), Black males relative to White males (S3b), and Black females relative to White females (S3c). The data highlight a consistent trend where Black males and females are less likely to be diagnosed with ADHD and more likely to be diagnosed with CD compared to White males and females, respectively. These findings underscore the significant sex and race disparities in the diagnosis of these disorders, with Pearson’s Chi-squared tests confirming a significant association between race and sex in the prevalence of ADHD and CD across all subtypes (Table S3).

## Discussion

ADHD is a complex neurodevelopmental disorder of varying presentations with social and cultural considerations. Our hypothesis that Black individuals are likely to have a lesser prevalence of an ADHD diagnosis and a higher prevalence of an ODD or CD diagnosis when compared to White individuals is supported by our findings. We demonstrate that ADHD diagnoses are overrepresented in White patients compared to Black patients and further reveal the impact of these diagnostic disparities across subtypes and sex. Apart from ADHD-HT, all other presentations of ADHD have significantly less prevalence in Black individuals than White individuals. The most notable difference was found to be in the diagnosis of ADHD-IT, where ADHD-IT is generally the most under-recognized and undertreated presentation of ADHD, with affected individuals having a low likelihood of receiving clinical and behavioral services^23^. This is particularly evident in minority populations, where cultural and societal factors may further complicate the diagnosis. Our results indicate that this problem may be compounded in the Black population, with disproportionately lower diagnoses of Black patients with ADHD-IT potentially contributing to significant disparities in access to clinical and behavioral services and utilization. Although the DSM-5 has introduced several changes aimed at improving ADHD diagnosis, such as raising the age of onset criterion and including examples for adult symptoms, inconsistencies in diagnosing ADHD subtypes persist. These include inconsistencies noted prior to the DSM-5 by Willcutt et al.^24^ and Valo and Tannock^25^, including variability in interpreting nuanced behaviors and symptom reporting tools.

Untreated ADHD-IT in adolescents and adults can be expected to impact an individual’s academic pursuits and career due to symptoms such as forgetfulness and difficulty maintaining attention for tasks, chores, or workplace responsibilities^23^. In Black children, ADHD symptoms can be disproportionately misconstrued as willful or defiant behaviors, contributing to a greater likelihood of a diagnosis of ODD or CD and a corresponding lack or mismatch of appropriate interventions or inappropriate use of disciplinary strategies^3,6,26^. A study using data from the 2011–2012 National Survey of Children’s Health found that White children were more likely to be diagnosed with ADHD alone, while Black children were more likely to be diagnosed with ADHD with an ODD or CD comorbidity^27^. Our findings add to the characterization of this disparity, demonstrating a significantly higher prevalence of ODD and CD diagnoses in all presentations between Black and White individuals, with a substantial increase in childhood-onset CD in the Black population. Typically, clinicians have demonstrated a reluctance to assign an early CD diagnosis for aggressive behavior in childhood, possibly expecting children to mature out of these patterns developmentally or, at times, giving an ODD diagnosis instead to avoid the stigma associated with a CD diagnosis^2^. However, our results indicate that this cautionary approach may not be afforded to Black children as often.

In addition to ADHD symptoms that persist from childhood into adulthood, there is a growing trend of adults presenting with inattention, disorganization, and impulsivity not recognized in childhood^28–30^. There is skepticism about the diagnosis of ADHD in adulthood, as it is not fully understood and may be driven by individuals seeking stimulant medication with the symptoms more likely explained by other psychiatric or substance use disorders^12,13,31^. Moreover, adult ADHD is not easy to identify. With the difficulty of obtaining a neuropsychiatric evaluation outside of childhood, especially for underprivileged populations, this job usually falls to primary care physicians (PCP) with little training in diagnosing complex psychiatric disorders^28,29^. The high prevalence of unspecified types of ADHD and CD in our cohorts indicates that PCPs might diagnose neurodevelopmental disorders without a closer examination of the diagnostic criteria necessary to identify the disorder presentation or possible comorbid conditions^28^.

While most ADHD diagnoses take place before the age of 18 in both populations, our analysis reveals that adult ADHD is diagnosed much more prominently in White adults compared to Black adults. The Black population has a steady decline in ADHD diagnoses after adolescence. However, in White patients, there is a disproportionately higher number of patients diagnosed between 18 and 40 than in Black patients. This difference may be due to implicit biases arising from societal or cultural disparities that could lead to the misinterpretation of service-seeking behaviors as stimulant-seeking actions. This misunderstanding may contribute to the underdiagnosis of Black adults with ADHD^7,30^.

Furthermore, this study goes beyond demonstrating race differences in diagnostic prevalence in ADHD and CD, we also reveal sex differences within each racial group in each subtype. Overall, females show a lower diagnostic prevalence in both ADHD and CD compared to their male counterparts, consistent with the literature that shows females are less likely to be diagnosed with neurodevelopmental disorders^5,16,32^. Yet, despite males displaying a higher prevalence of ADHD and CDs, females generally suffer from more severe symptoms, significant lifetime psychiatric comorbidities, and functional impairments^5,16,32^. Apart from diagnostic bias, a potential contributing factor to sex disparities in these disorders is variation in symptom presentation. Females tend to exhibit more inattentive behavior and less hyperactivity, making them perceived as less disruptive than males, and their ADHD might go unnoticed or undiagnosed^33,34^. As for CD, while males are inclined to display signs of proactive physical aggression, females are more likely to show relational, reactive aggression in bullying and manipulative behavior with less callous-unemotional traits^32^. Beyond developmental sex differences, this could also suggest that females must exhibit more pronounced symptoms before being referred for or diagnosed with these disorders. Our analysis further demonstrates that Black females are the least prevalent group diagnosed with all presentations of ADHD. Additionally, apart from predominantly inattentive ADHD, White females are the second most underrepresented group in ADHD diagnoses. White females also appear to be the group least diagnosed with CDs.

Prior studies have revealed that conscious or unconscious bias can impact medical decisions and diagnoses^6^. These studies indicate clinicians are more responsive to non-Hispanic White patients seeking an ADHD diagnosis and treatment, whereas Black students receive fewer referrals from schoolteachers and administrators^5,6^. Moreover, obtaining a CD diagnosis will likely negatively impact a caregiver’s ability to detect inattentive or hyperactive behavior, limiting their access to psychiatric evaluations, medication, and therapy^6^. It could also lead to harsher disciplinary measures and exclusionary practices in school that could further compound mental and behavioral challenges^5,6^. Furthermore, cultural factors can significantly impact what families share with clinicians regarding their children’s emotional and behavioral problems. In many minority communities, norms and values influence perceptions of mental health, often leading to underreporting of symptoms. Mistrust of medical providers, stemming from historical injustices and negative healthcare experiences further exacerbates this issue, resulting in hesitancy to seek medical help and underreport symptoms. Culturally sensitive approaches in clinical practice are essential to address these disparities. Training in cultural competence can help mitigate biases and improve diagnostic accuracy across diverse populations.

One concern related to overdiagnosis and overtreatment of neurodevelopmental disorders like ADHD is the potential harm stemming from diverting resources from other populations who may be underdiagnosed or undertreated^35^. Furthermore, the perception of ADHD as an overdiagnosed disorder in any racial group is likely^36^. Future studies should aim to investigate other racial and ethnic minorities, such as those from indigenous and Hispanic backgrounds.

Another limitation of this study is that we did not have access to robust information about our patient cohorts’ socioeconomic status and insurance status, which is often a factor in a patient’s ability to receive clinical care, particularly mental healthcare and treatment^5^. Furthermore, with our reliance on ICD-10 codes, there were no symptom details to determine diagnostic accuracy. Third, the lower prevalence of ADHD in the TriNetX database, compared to population-based estimates, may reflect selection bias due to the reliance on clinical diagnoses recorded in healthcare settings, which could miss undiagnosed or untreated cases. This bias highlights the need for cautious interpretation of the findings and further research to ensure more representative data collection methods.

Overall, our analysis found race and sex disparities in ADHD and CD diagnosis across associated subtypes in a large-scale US dataset. The non-Hispanic Black population is less likely to be diagnosed with ADHD but more likely to be diagnosed with ODD or CD than the non-Hispanic White population. White patients get diagnosed with ADHD in adulthood more often than Black patients. Black females are the cohort least likely to be diagnosed with ADHD, and White females are the cohort least likely to be diagnosed with CD. Future work that integrates patients’ socioeconomic status and insurance status will give a deeper understanding of racial disparities. Detailed clinical symptom presentations could be used to analyze further and measure accurate rates of over- and under-diagnosis in suspected populations. The disproportionally high rates of ODD and CD diagnoses carried by Black patients may indicate unconscious/implicit bias by healthcare practitioners and a corresponding tendency to miss underlying conditions that could better explain disruptive behaviors. Presenting evidence and increasing awareness of such disparities has effectively reduced unconscious bias and sustained the movement toward more culturally informed and objective psychiatric evaluations^6^.

## Supplementary Information

Supplementary Information.

## Data Availability

All data used in the present study is available through TriNetX at https://trinetx.com/
