# Supplementary material for: Large-scale analysis reveals racial disparities in the prevalence of ADHD and conduct disorders
Source: Sci Rep. 2024 Oct 24;14:25123. doi: 10.1038/s41598-024-75954-5 (PMC11502815; doi:10.1038/s41598-024-75954-5)
Supplement: Supplementary file 1 — Supplementary Information. [file 41598_2024_75954_MOESM1_ESM.pdf]

Figure S1: Prevalence Ratios of ADHD and CD Between Black and White individuals. Prevalence ratio of A. ADHD and its presentations, and B. CD and its presentations in Black and White populations. ADHD; Black N = 141,277, White N = 708,004; CD; Black N = 47,437, White N = 110,160. *p < 0.01, ***p < 0.0001

A.

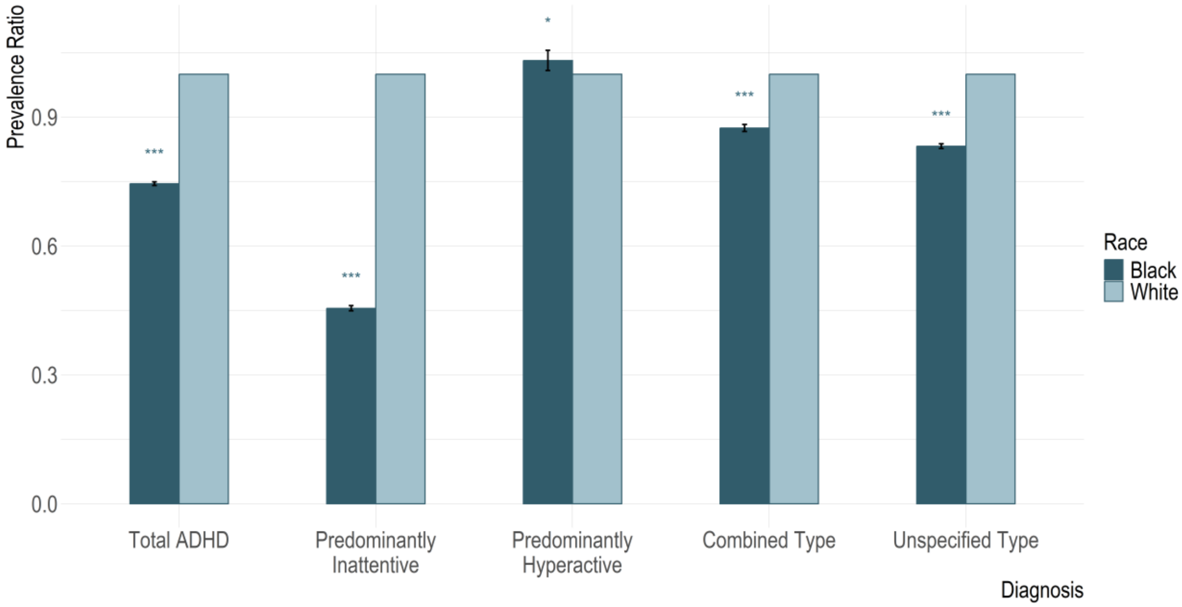

B.

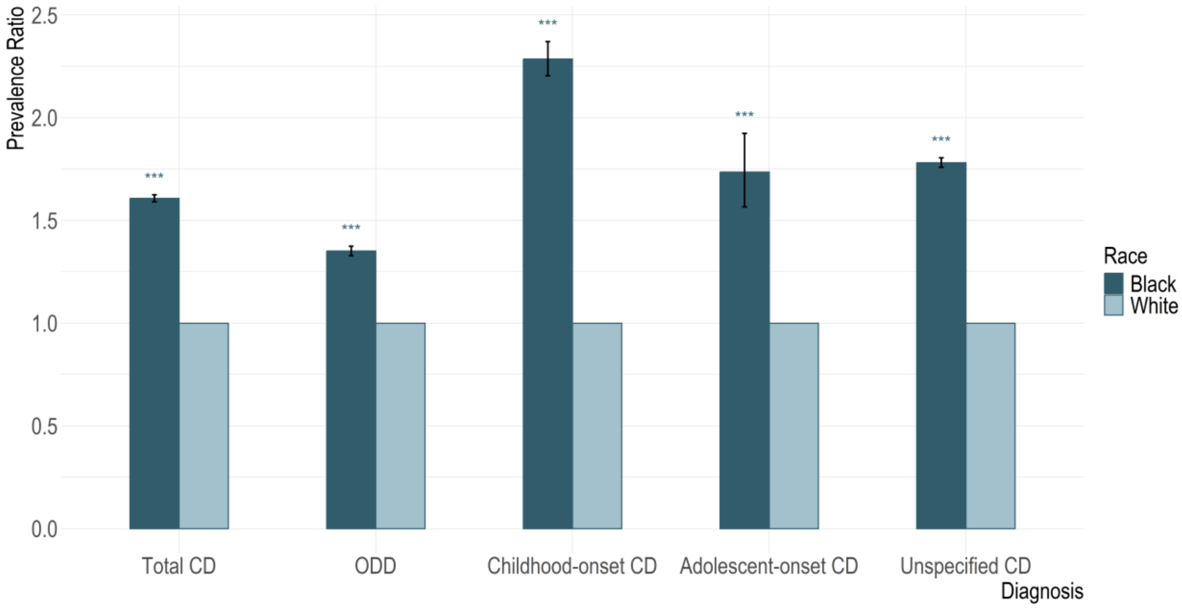

Figure S2: Sex Differences in ADHD and CD Prevalence Ratios Between Black and White individuals. Prevalence ratio of A. ADHD and its presentations and B. CD and its presentations in males and females in Black and White populations. ADHD; Black Female N = 51,323, Black Male N = 89,935, White Female N = 313,138, White Male N = 394,607; CD; Black Female N = 17,047, Black Male N = 30,381, White Female N = 36,300, White Male N = 73,837. ***p<0.0001

A.

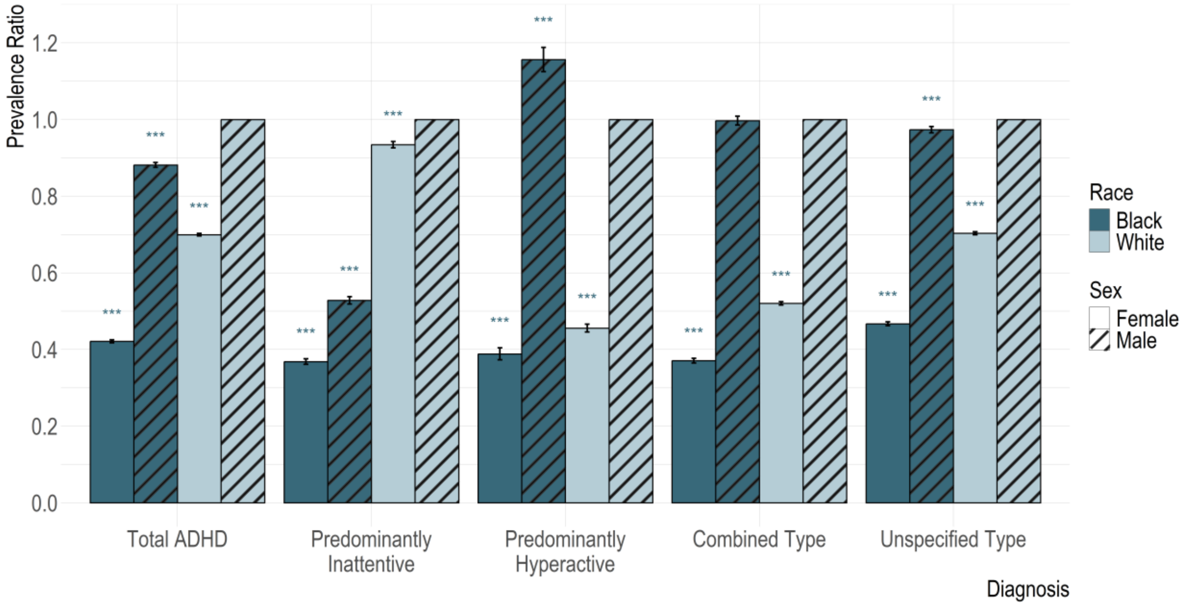

B.

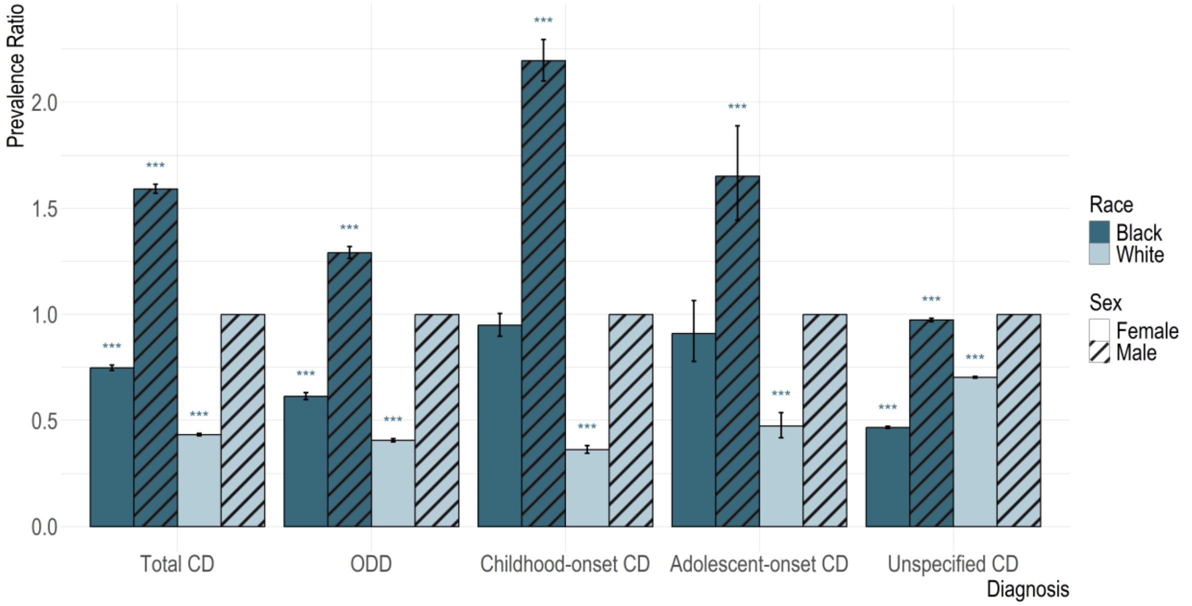

Figure S3: Odds Ratio and 95% confidence interval of the prevalence of ADHD and CD and their presentations in **A.** Black females compared to White males, **B.** Black males compared to White males, and **C.** Black females compared to White females.

A.

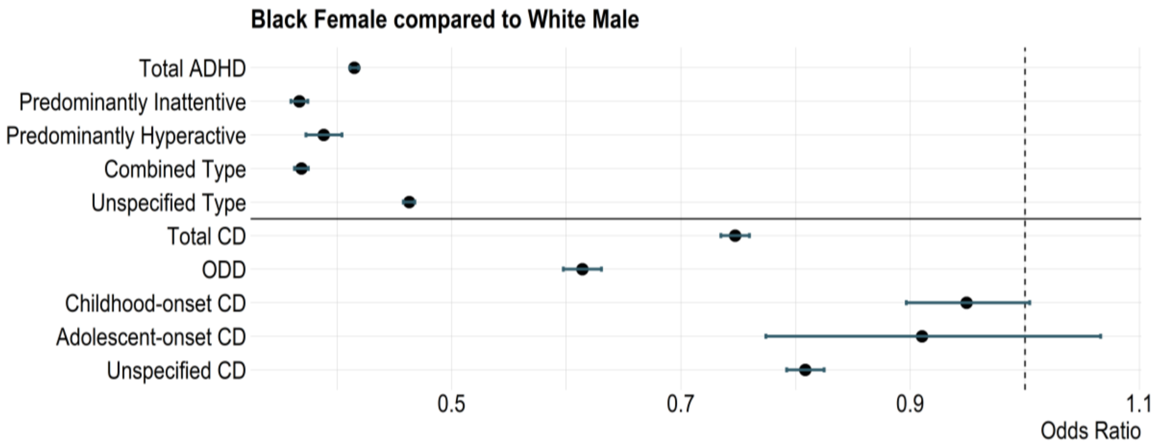

B.

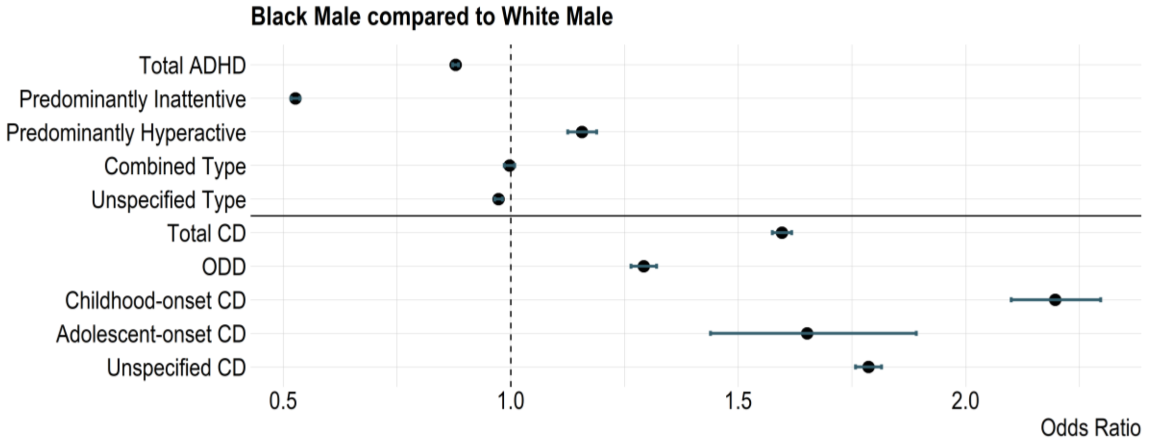

C

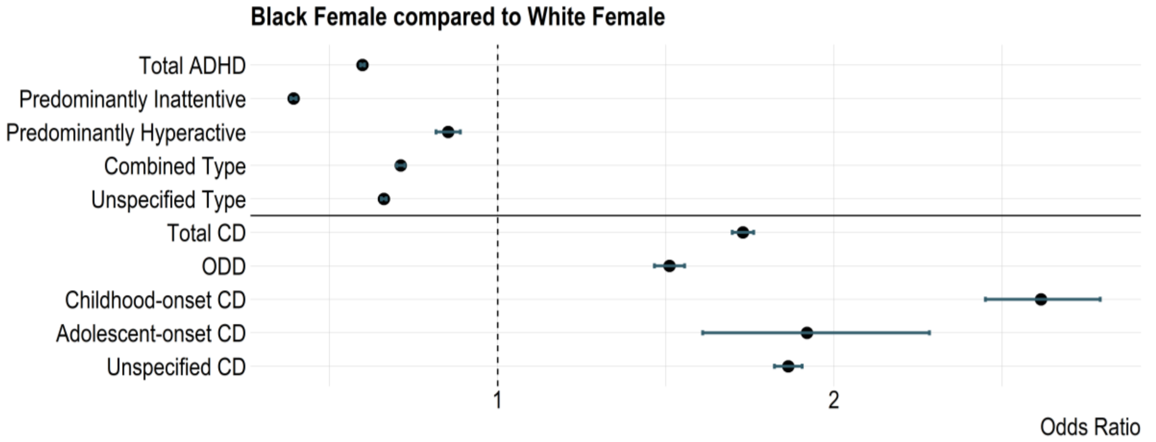

Table S1: Race and sex distribution of the patient population

	Number of patients in group	%	Number of ADHD patients	%	Number of CD patients	%
Race						
Black/African American	8,702,848	21.1	141,277	1.62	47,437	0.55
Sex						
Female	4,654,839	53.5	51,323	36.3	17,047	35.9
Male	3,899,810	44.8	89,935	63.7	30,381	64.0
White	32,489,776	78.9	708,004	2.18	110,160	0.34
Sex						
Female	17,111,169	52.7	313,138	44.2	36,300	33.0
Male	15,083,418	46.4	394,607	55.7	73,837	67.0

Table S2: Statistical differences in the ages of first diagnosis of ADHD and CD between black and white patients

	Black	White	p-value
ADHD			
Mean	15.7	23.9	<0.0001
Range	1 - 88	1 - 108	
ADHD (Children)			
Mean	9.00	9.51	<0.0001
Range	1 - 18	1 - 18	
CD			
Mean	10.5	10.3	<0.0001
Range	1 - 25	1 - 25	

Table S3: ADHD and CD Pearson's Chi-squared test

		Black	White	χ^2	p-value
ADHD					
	Female	51,323	313,138	3008.5	<0.0001
	Male	89,935	394,607		
CD					
	Female	17,047	36,300	131.81	<0.0001
	Male	30,381	73,837		
